# Positional treatment without mechanical ventilation in a very preterm infant with unilateral pulmonary interstitial emphysema: case report and review of the literature

**DOI:** 10.1186/s12887-019-1640-2

**Published:** 2019-08-01

**Authors:** Xiaoping Lei, Oliver Stangl, Christina Bösche, Kristina Stuchlik, Roland Czorba, Christian Wieg

**Affiliations:** 1 Department of Neonatology, Affiliated Hospital of Southwest Medical University, Luzhou, Sichuan China; 2 Department of Neonatology and Pediatric Intensive Care, Klinikum Aschaffenburg, Am Hasenkopf 1, Aschaffenburg, 63739 Aschaffenburg, Bavaria Germany; 3 Department of Gynecology and Obstetrics, Klinikum Aschaffenburg, Aschaffenburg, Germany

**Keywords:** Pulmonary interstitial emphysema, Non-invasive treatment, Prematurity

## Abstract

**Background:**

Pulmonary interstitial emphysema (PIE) in very low birth weight infants is a rare but severe complication. Although most of these air leaks develop in mechanically ventilated infants, they have also been reported in infants exposed only to nasal continuous positive airway pressure (CPAP). The optimal treatment for PIE is still under discussion and includes different approaches such as unilateral intubation, high frequency oscillation ventilation and even surgical lobectomy. However, as yet, there has been no report on complete resolution of unilateral PIE by positioning therapy without mechanical ventilation.

**Case presentation:**

We report the case of a 28^+1^gestational week twin, 990 g birth weight, Apgar 9–10-10. After stabilization with nasal CPAP the baby received surfactant by less invasive surfactant application (LISA) technique in the delivery room after 35 min of life, and continued respiratory support with nasal CPAP. At day 5 X-ray presented unilateral PIE, while pCO_2_ increased from 40 mmHg to 55 mmHg and FiO_2_ from 0.21 to 0.28 to achieve SpO_2_ in the target range of 89–94%. The baby was treated by strict positioning on the affected hemithorax in a special splint while spontaneously breathing on High Flow Nasal Cannula (HFNC). Complete resolution of the unilateral PIE was observed after 96 h. No chronic lung disease developed.

**Conclusion:**

For unilateral PIE in very preterm infants, positioning on the affected hemithorax without mechanical ventilation is a therapeutic option.

## Background

Pulmonary interstitial emphysema (PIE) is a well known and severe complication in very premature newborns with respiratory distress syndrome (RDS) receiving invasive mechanical ventilation. However, few PIE cases - unilateral or bilateral - have been reported in premature babies exposed to nasal continuous positive airway pressure (nCPAP) [1–7]. So far, there is no standard treatment for unilateral PIE, and several treatment approaches have been reported in literature, including selective unilateral intubation and ventilation [8–12], High Frequency Oscillation Ventilation (HFOV) and High Frequency Jet Ventilation (HJFV) [12–16], Neurally Adjusted Ventilatory Assist (NAVA) [17, 18], and even surgical lobectomy [19–21]. Positioning of the baby on the affected side is considered an adjunct to these therapies [12, 15, 22, 23]. However, as yet, there is no report on complete resolution of unilateral PIE by positioning therapy without mechanical ventilation.

We report on a very low birth weight (VLBW) infant who developed unilateral PIE secondary to nasal CPAP. The baby was treated by a strict positioning on the affected hemithorax in a special splint while spontaneously breathing on high-flow nasal cannula (HFNC). PIE completely resolved in 96 h. Considering the reported interventions in literature, our aim is to add to the available knowledge on therapy concepts for unilateral PIE.

### Case presentation

**Fig. 1:**
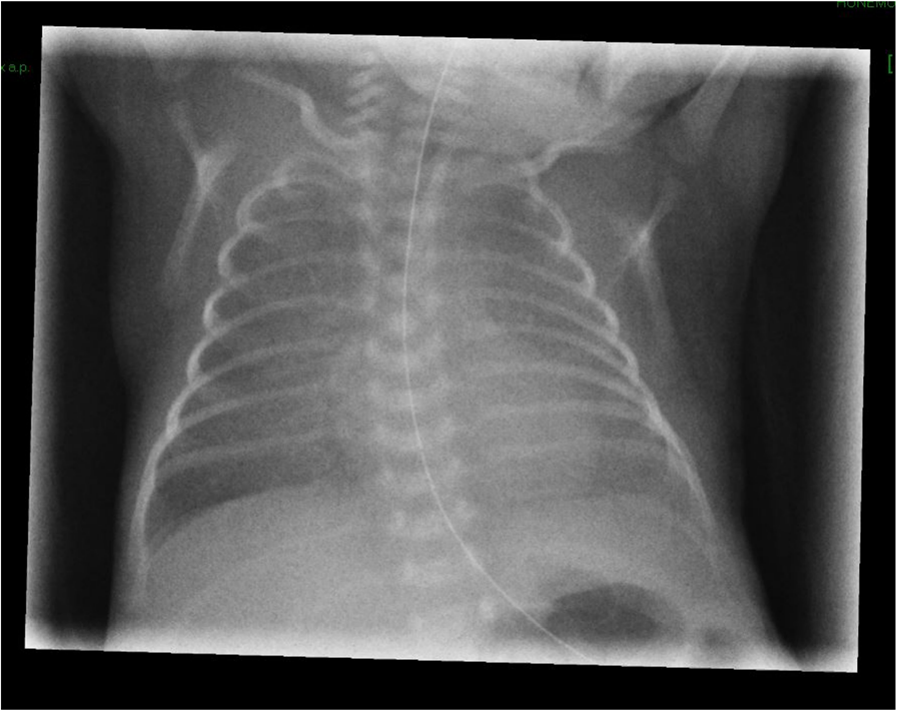
Initial X-ray chest: respiratory distress syndrome grade 1°. One hour after LISA. Nasal CPAP settings: 6.5 mbar, FiO_2_ 0.21

Second twin, born after 28^+ 1^ weeks of gestation, 990 g-birth weight, Apgar 9–10-10. Steroid prophylaxis was given to the mother 96 h before C-Section was performed due to onset of labour and premature rupture of the membranes. No clinical symptoms, laboratory, or histological signs of chorioamnionitis were detected. After stabilization with nasal CPAP (FiO_2_ 0.4, Silverman score 4) the baby received surfactant by less invasive surfactant application (LISA) procedure in the delivery room 35 min of after birth. Initial X-ray (one hour after LISA) showed RDS 1° (Fig. 1). After good response to surfactant, respiratory support with nasal CPAP (5.5 to 6.5 mbar) was continued for the following 5 days with FiO_2_ 0.21 for SpO_2_ 89–94%. PCO_2_ ranged between 40 and 50 mmHg and the Silverman Score was 1–2. The pressure levels of nasal CPAP were 6.5 mbar for the first 8 h after LISA, followed by 6.0 mbar for another 12 h and 5.5 mbar from 2nd to 5th day. A second X-ray at the age of 24 h showed regression of RDS and bilateral symmetrical ventilation of the lung, without signs of over-distension or air leak. Thus, unilateral surfactant delivery could be ruled out.

**Fig. 2:**
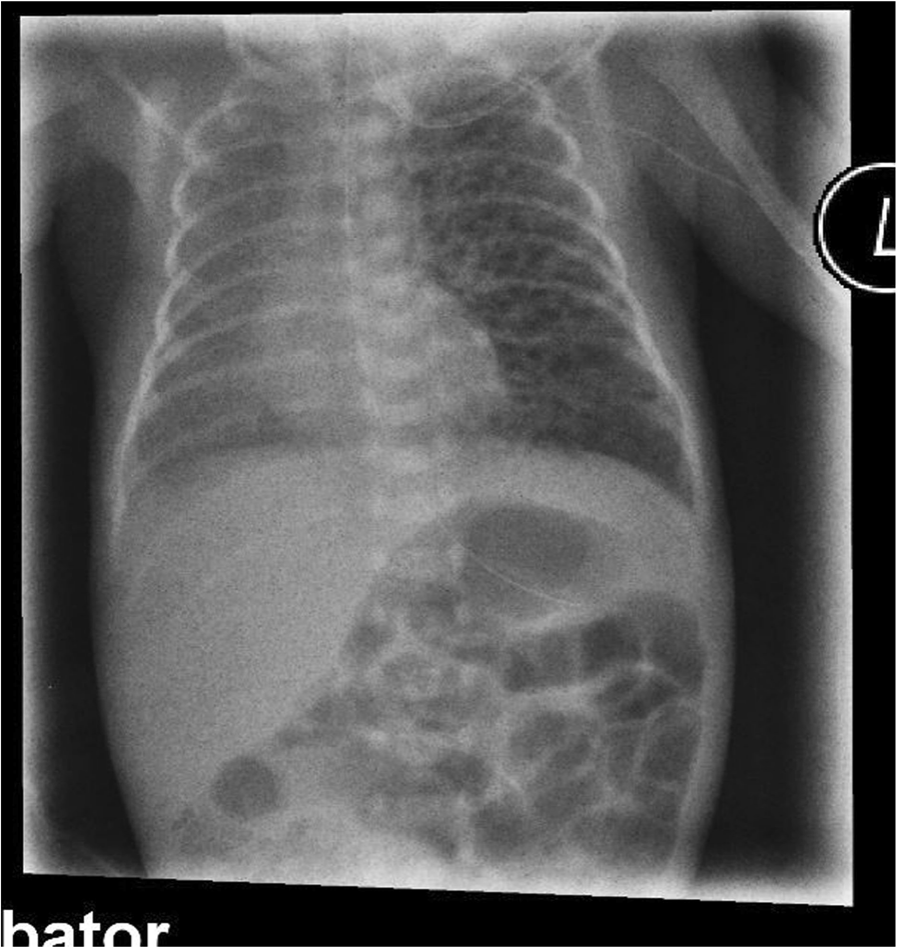
X ray chest day 5 of life: Severe left unilateral pulmonary interstitial emphysema. Left-sided diffuse pulmonary interstitial emphysema, mild mediastinal shift to the right. Nasal nCPAP settings: 5.5 mbar, FiO_2_ 0.28

**Fig. 3:**
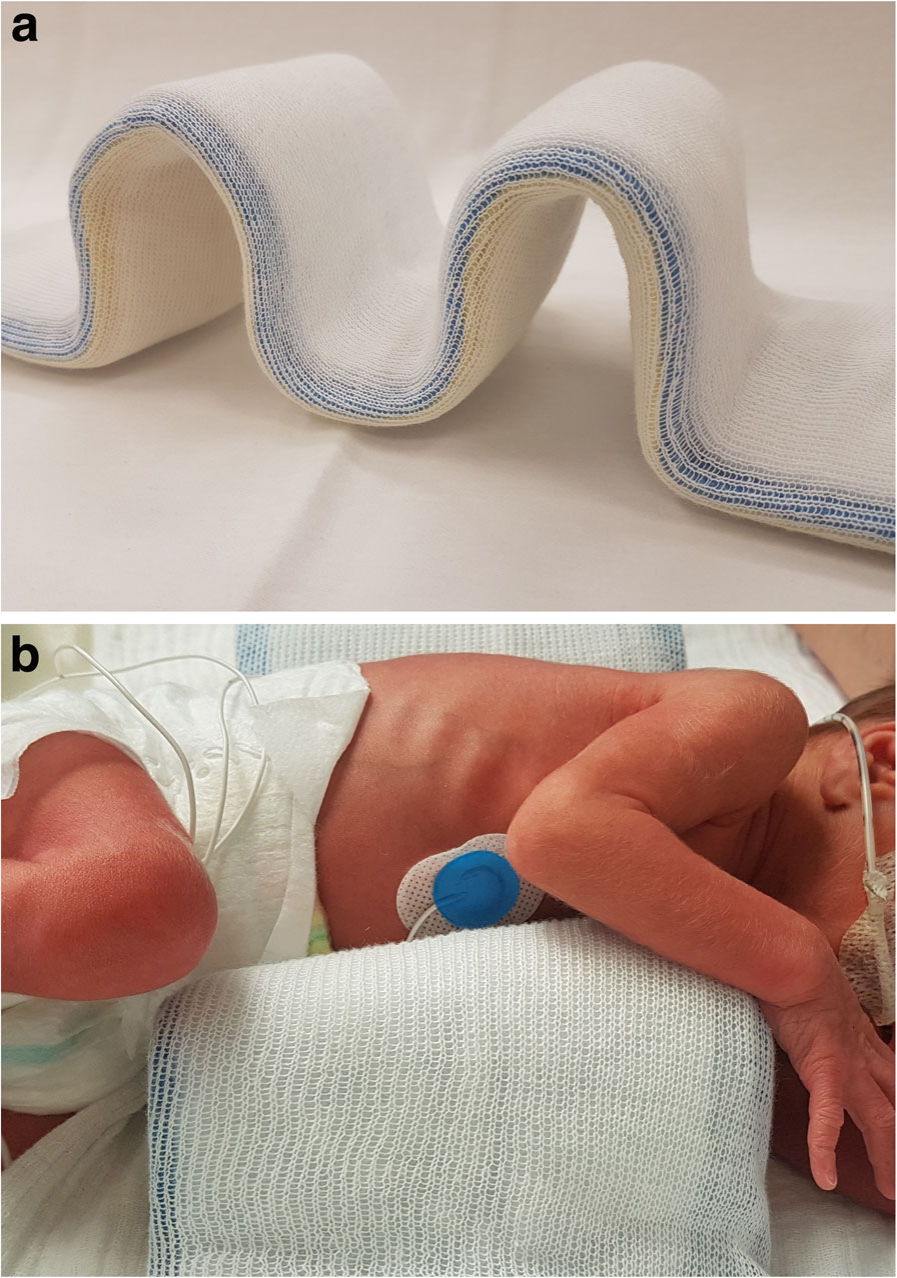
**a** The splint used for Positioning Therapy. **b** Positioning Therapy in the splint

**Fig. 4:**
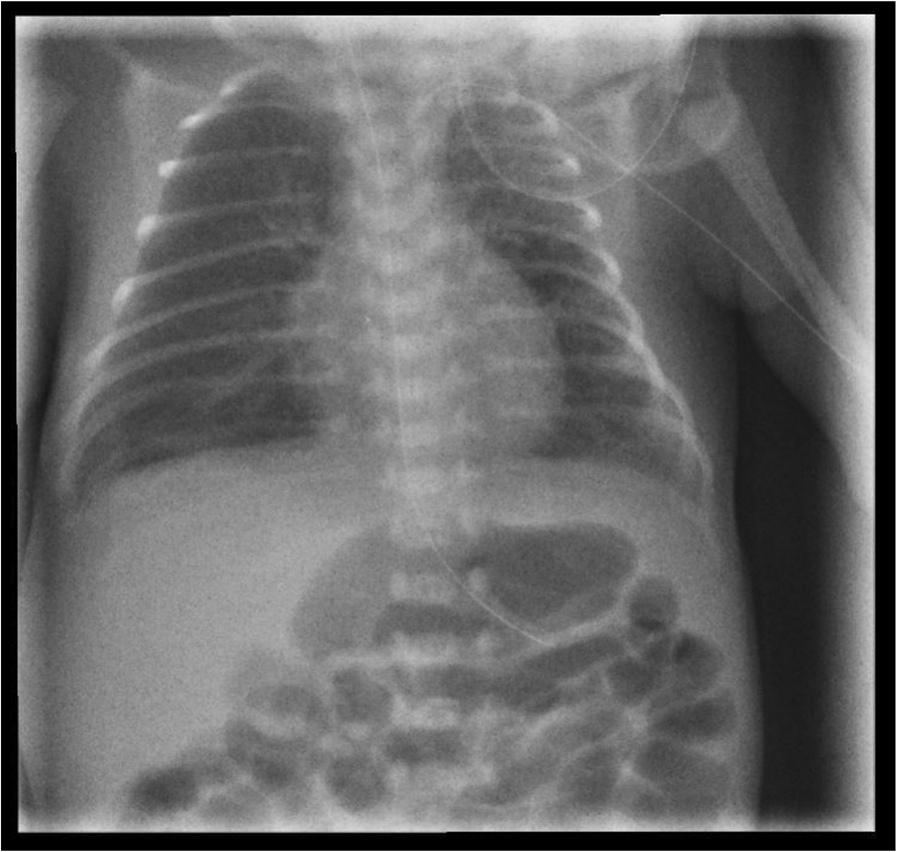
X ray chest day 9 Resolved Unilateral Pulmonary Interstitial Emphysema. No cysts are presented in the left lung, mediastinal shift recovered. High-flow nasal cannula settings: Flow 6 L/min, FiO_2_ 0.21–0.25

**Fig. 5:**
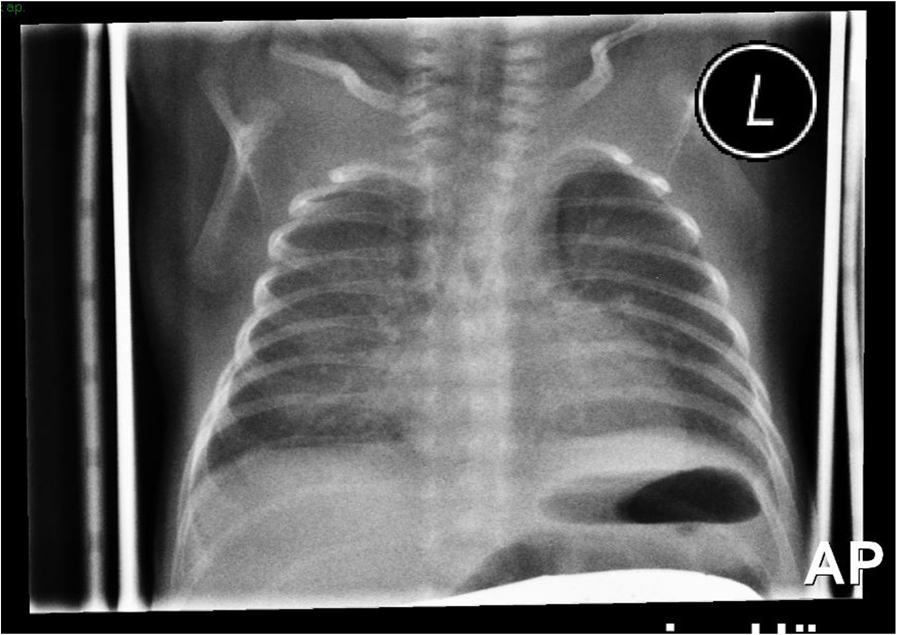
X ray chest 10 weeks of life: No significant signs of bronchopulmonary dysplasia 10 weeks after birth. Bilateral central opaque zones are visible, the baby didn’t need oxygen or any respiratory support for SpO2 > 92%

At day 5, pCO_2_ increased from 40 to 55 mmHg and FiO_2_ from 0.21 to 0.28 for SpO_2_ 89–94% was needed, while apnea–bradycardia syndrome was more pronounced (minimal heart rate 75/min). The Silverman Score rose to 3–4. A sepsis was ruled out by negative Il-6 and CRP, and blood pressure measurements were normal. X-ray indicated severe left unilateral PIE, shifting the mediastinum to the right (Fig. 2). We changed nasal CPAP to High-Flow Nasal Cannula (HFNC) with flow 6 l/min, used a 1 mm bi-nostril prong-interface and a small pacifier to keep the mouth close, by calculated pressure 3.0–4.0 mbar and performed a strict positioning therapy in a splint (Fig. 3a and b) on the affected left hemithorax. This stiff, but pliant padded plastic splint is normally used for the stabilization of forearm bone-fractures in the Emergency Room. Mild hypercapnia (paCO_2_ 60 mmHg) occurred and FiO_2_ was needed up to 0.4 to reach target SpO_2_ of 88–92%. Following unilateral positioning in the splint, the respiratory status improved, FiO_2_ was gradually decreased from 0.4 to 0.25, and complete resolution of PIE was demonstrated by X-ray after 96 h (Fig. 4). We continued HFNC with FiO_2_ between 0.21 and 0.25 up to day 35 (33^+ 2^ gestational week). When the baby was discharged after 10 weeks, SpO_2_ was 99% (Table 1). Though a few bilateral central opaque zones were visible, significant signs of bronchopulmonary dysplasia (BPD) were not detectable on X-ray (Fig. 5). Several ultrasound examinations of the brain were performed including one at discharge. We never detected any signs of Intraventricular Haemorrhage (IVH) or any stages of periventricular Leukomalacia (PVL).

**Table 1:** The Clinical Parameters of this Case: From Birth to Discharge

	Before LISA (0-35 min)	The first day after LISA	2nd-5th day	Onset PIE (5th day)	Begin positioning therapy	5th–9th day	10th - 35th day	To discharge
Ventilation	n CPAP	n CPAP	n CPAP	n CPAP	HFNC	HFNC	HFNC	No
Target SpO_2_%	89–94	89–94	89–94	89–94	89–92	89–92	90–95	92–99
FiO_2_%	0.4	0.21	0.21	0.28	0.4	From 0.4 to 0.25	From 0.25 to 0.21	air
Silverman Score	3	1–2	0–1	3–4	1–3	1–2	0–1	0–1
pCO_2_ mmHg	35	40	40–50	53	60	40–55	45–55	40–55
Pressure mbar	6.5	6.5–6.0	5.5	5.5	3–4 (calc)	3–4(calc)	2–3(calc)	None

*CPAP* continuous positive airway pressure, *HFNC* High Flow Nasal Cannula, *LISA* less invasive surfactant application, *PIE* pulmonary interstitial emphysema

## Discussion and conclusions

This case demonstrates that unilateral PIE developed in a spontaneously breathing baby, may resolve by positioning the baby on the affected hemithorax in a special splint without mechanical ventilation.

PIE is a life-threatening form of air leak syndrome in very premature infants with RDS. It also occurs in Meconium-Aspiration-Syndrome in term-infants. Its pathophysiology was firstly discussed by Macklin [24] in 1939. When infants are exposed to positive pressure ventilation, especially - but not exclusively - while intubated and mechanically ventilated, potential loss of the epithelial integrity of the small airways, sacculi or immature alveoli permits entering air into the perivascular tissue of the pulmonary interstitium. By spreading into the bronchovascular bundles, it may result in pneumothorax, pneumomediastinum, pneumopericardium, pneumoperitoneum, subcutaneous emphysema, and terminally massive air embolism [24, 25]. The entrapment of air can initiate compression of functional lung tissue and vascular structures, resulting in adjacent atelectasis, leading to severe impairment of lung function and increased respiratory distress of the baby.

Prematurity, meconium aspiration resuscitation, and hyperoxia are known to be risk factors for PIE [26–28]. A high risk for air leaks including PIE seems to be surfactant deficiency in mechanical ventilated very premature babies not treated with surfactant [27, 29]. A study from the pre-surfactant era, the incidence of PIE in ventilated preterm infants is reported up to 32% [27]. Technical risk factors include use of high peak inspiratory pressure (PIP) and/or high positive end expiratory pressure (PEEP) in mechanical ventilation, high (FiO2), long inspiratory time and large tidal volumes [1, 25–27, 30]. Malpositioning of the tube in one bronchus may also be responsible for unilateral PIE [30].

In our case, the baby received LISA followed by nasal CPAP, exposed to widely accepted pressure levels in the delivery room, avoiding mask or mechanical ventilation. This case is consistent with a previous study that indicates the incidence of PIE reaches 11.5% in cases receiving nasal CPAP only and/or non-invasive ventilation in delivery room management [1]. The X-ray on the first day (Fig. 1) shows symmetrical lung ventilation ruling out the main differential diagnoses unilateral surfactant delivery, adenocystic lung malformation and congenital lobe emphysema.

When PIE is treated successfully, secondary serious complications can be prevented. In older reports, fatality rates of premature infants with PIE reach 80% [31] and recent data still show a high risk of death (37%) [28]. In survivors, high proportions of adverse outcomes with significant morbidity such as IVH (54%) and BPD (77%) are reported [1, 32].

The generally accepted treatment strategy for PIE includes maintaining low levels of peak inspiratory pressure, mean airway pressure and low tidal volumes, accepting high FiO_2_ and moderate hypercapnia. Regarding our case, unilateral PIE resolved completely by positional treatment and reduction of the calculated airway pressure. Previous studies from the 1980’s [22, 23] reported that positioning the baby laterally on the affected hemithorax for several hours, while ventilating with low airway pressure achieved ideal results in neonates with unilateral PIE.

In 1988, Gortner et al. [15] reported on a concept of positioning therapy combined with HFOV which proved remarkable successful. By positioning the baby on the affected side, the mediastinum, following gravity, can compress the lung of the underlying hemithorax, while the unaffected lung is still ventilated. To keep the baby in the right position we used a splint as described above. The soft, padded surface protects the vulnerable skin of the baby. Fixing spontaneously breathing premature infants without sedation in a lateral position may be challenging, so the splint provides an effective significant support. We here demonstrate that positioning therapy can be successful for babies receiving HFNC also.

Using a three-dimensionally printed airway model a recent study [33] demonstrated that, while the baby’s mouth closed, a flow of 6 l/min in HFNC can achieve pressures of approximately 4.0 mbar in preterm infants. HFNC also provides active CO_2_ out-washing from the nasopharynx [33]. In view of these potential advantages we selected this mode during positioning therapy. It should be emphasized, that there are no data available comparing HFNC versus nCPAP in unilateral PIE. Thus, the mode of gas delivery may not have been as important as the positioning therapy in our case.

The challenge in the treatment of unilateral PIE is to find the balance between beneficial therapeutic approaches for the affected lung and the potential harmful side effects for the contralateral lung. Selective ventilation of the non-affected lung has been advocated as it provides the possibility of reabsorption of interstitial air by reducing the alveolar pressure of the affected lung to zero. Atelectasis and ventilator-induced lung injury of the ventilated lung were reported as the limitations of selective ventilation [8]. High frequency ventilation has also been used for PIE to attain an adequate gas exchange with lower mean airway pressures and volume than conventional ventilation [13]. However, HFOV itself contributes to PIE [25] and the low volume strategy induce alveolar hypoventilation of the unaffected lung, leading to severe hypercapnia and dependency on high FiO_2_.

In our case, strictly performed positioning on the affected hemithorax, combined with HFNC, seems to be a successful alternative to more invasive strategies in the treatment of unilateral PIE. The use of a flexible splint provides the possibility to maintain the baby in the required position. This approach may offer a good chance to reduce invasive ventilation, and if tolerated, it should be considered to be the first line treatment. However, it must be emphasised that infants who continue to have respiratory acidosis and/or significant work of breathing will require other types of respiratory support.

## Data Availability

All the important information of this case are included in the manuscript.

## Abbreviations

BPD: Bronchopulmonary dysplasia
CPAP: Continuous positive airway pressure
HFJV: High Frequency Jet Ventilation
HFNC: High Flow Nasal Cannula
HFOV: High Frequency Oscillation Ventilation
IVH: Intraventricular haemorrhage
LISA: Less invasive surfactant application
NAVA: Neurally Adjusted Assist Ventilation
PEEP: Positive end-expiratory pressure
PIE: Pulmonary interstitial emphysema
PIP: Peak inspiratory pressure
PVL: Periventricular leucomalacia
RDS: Respiratory distress syndrome
